# I See What You Are Saying: Hearing Infants’ Visual Attention and Social Engagement in Response to Spoken and Sign Language

**DOI:** 10.3389/fpsyg.2022.896049

**Published:** 2022-06-30

**Authors:** Miriam A. Novack, Dana Chan, Sandra Waxman

**Affiliations:** ^1^Department of Medical Social Sciences, Feinberg School of Medicine, Northwestern University, Chicago, IL, United States; ^2^Department of Psychology, Northwestern University, Evanston, IL, United States

**Keywords:** spoken language, sign language, infants, categorization, multimodal

## Abstract

Infants are endowed with a proclivity to acquire language, whether it is presented in the auditory or visual modality. Moreover, in the first months of life, listening to language supports fundamental cognitive capacities, including infants’ facility to form object categories (e.g., dogs and bottles). Recently, we have found that for English-acquiring infants as young as 4 months of age, this precocious interface between language and cognition is sufficiently broad to include not only their native spoken language (English), but also sign language (American Sign Language, ASL). In the current study, we take this work one step further, asking how “sign-naïve” infants—hearing infants with no prior exposure to sign language—deploy their attentional and social strategies in the context of episodes involving either spoken or sign language. We adopted a now-standard categorization task, presenting 4- to 6-month-old infants with a series of exemplars from a single category (e.g., dinosaurs). Each exemplar was introduced by a woman who appeared on the screen together with the object. What varied across conditions was whether this woman introduced the exemplar by speaking (English) or signing (ASL). We coded infants’ visual attentional strategies and their spontaneous vocalizations during this task. Infants’ division of attention and visual switches between the woman and exemplar varied as a function of language modality. In contrast, infants’ spontaneous vocalizations revealed similar patterns across languages. These results, which advance our understanding of how infants allocate attentional resources and engage with communicative partners across distinct modalities, have implications for specifying our theories of language acquisition.

## Introduction

Infants are endowed with a proclivity to acquire language (Kuhl, 2000). Importantly, this propensity is not restricted to a single modality: infants are prepared to acquire any human language, whether it is spoken or signed (Meier and Newport, 1990; Bavelier et al., 2003; Petitto et al., 2004; Pichler, 2011; Newport and Meier, 2017). Even without exposure to sign language, infants prefer looking at sign language over non-linguistic hand movements (Krentz and Corina, 2008) and are sensitive to its linguistic features (Baker et al., 2006; Palmer et al., 2012; Stone et al., 2018). However, for infants who are only exposed to spoken language, early sensitivity to sign language wanes over the first year of life (Baker et al., 2006; Krentz and Corina, 2008; Palmer et al., 2012; Stone et al., 2018). Infants’ natural tendency to acquire language is thus flexible with respect to modality but is rapidly attuned to the language modality of the linguistic communit(ies) that surround them.

Infants’ preference for language also has powerful downstream consequences. For hearing infants as young as 4 months of age, listening to infant-directed speech modulates neural activity in such a way as to engage early attentional components (Woodruff Carr et al., 2021). In addition, listening to language supports infants’ fundamental cognitive capacity to form object categories (Waxman and Markow, 1995; Balaban and Waxman, 1997; Waxman and Braun, 2005; Ferry et al., 2010). Evidence for this early emerging interface between language and cognition comes from a robust paradigm, in which infants are familiarized to a series of exemplars, all from the same category (e.g., dinosaurs). What varies is whether these exemplars are introduced in conjunction with infant-directed speech (e.g., “look at the modi”) or with well-matched non-linguistic sounds (e.g., sine-wave tones, backward speech). At test, infants then view two new exemplars: one from the now-familiar category (e.g., a new dinosaur) and another from a novel category (e.g., a fish). If infants form the object category during familiarization, they should distinguish the novel from the familiar category objects at test. The results reveal that for infants from 3 to 12 months, listening to language confers a cognitive advantage: Infants who hear infant-directed speech in conjunction with familiarization exemplars successfully form object categories, whereas infants who see the same exemplars paired with non-linguistic acoustic signals do not (Waxman and Markow, 1995; Balaban and Waxman, 1997; Waxman and Braun, 2005; Fulkerson and Waxman, 2007; Ferry et al., 2010, 2013). This early link between language and cognition provides a foundation for learning and becomes increasingly precise with development (Perszyk and Waxman, 2018).

In recent work we asked whether this precocious link is sufficiently abstract to include language presented in the visual modality (Novack et al., 2021). Focusing on 4- to 6-month-old hearing infants with no prior exposure to sign language, we adapted the categorization task described above, this time pairing each familiarization object with a woman who communicated about the object in one of two ways. In a non-linguistic condition, she pointed at the object, and looked back and forth between the object and the infant, providing social-communicative pedagogical cues but no linguistic information. In a sign language condition, she signed the phrase “LOOK MODI, YOU SEE MODI?” in American Sign Language (ASL), together with the same pointing and eye-gaze cues presented in the non-linguistic condition.

The results were straightforward: At 4 months, infants in the sign language condition—but not the non-linguistic condition—successfully formed object categories (Novack et al., 2021). By 6 months, this advantage had waned: infants failed to form object categories in either condition. This developmental tuning is consistent with evidence that between 4 and 6 months, infants rapidly narrow the range of signals that they will link to cognition, a narrowing that is shaped by the language(s) in which they are immersed (e.g., Ferry et al., 2013; Perszyk and Waxman, 2018, 2019).

One key feature of the design used in Novack et al. (2021), which we retain in the current study, is worth noting: this was the first study of its kind in which the communicative partner was visible, engaging the infant from the screen. This is an important departure from prior instantiations of the object categorization task in which objects were presented visually, and the linguistic and non-linguistic information was presented acoustically (e.g., Waxman and Markow, 1995; Balaban and Waxman, 1997; Waxman and Braun, 2005; Fulkerson and Waxman, 2007; Ferry et al., 2010, 2013). Necessary to study infants’ responses to sign language, this design shift also provides the unique opportunity to examine the broader matter of how infants integrate multiple sources of information (the images of objects and the language input to describe them) when presented within a single modality.

Here, we advance the prior design to focus on infants’ visual attentional and social engagement strategies in the context of observing either sign language or spoken language. Moving beyond object categorization as an outcome measure, we focus instead on infants’ engagement during learning, as they view a series of objects, each accompanied by a woman who introduces each object in either ASL or in spoken English. At issue is whether infants (i) deploy different visual attentional strategies, and/or (ii) adopt different social engagement strategies, in the context of either spoken versus sign language.

Indeed, there are good reasons to expect that infants’ engagement may differ when presented with sign language or spoken language. Consider, for example, the case of object labeling. Infants acquiring spoken language can devote their full visual attention to the object under description, as they receive the linguistic information through the auditory channel. In contrast, infants acquiring sign language must divide their visual attention strategically between the object and a signer.

In designing our measures, we took advantage of compelling evidence that young children who are exposed to sign language do indeed divide their visual attention strategically and fluidly between a signer and a referent object during word-learning episodes. For instance, sign-exposed toddlers assess the structure of linguistic input to advantageously allocate their visual attention between a signer and a referent when fast-mapping novel signs (Lieberman et al., 2021) or when finding a known referent (MacDonald et al., 2020). They also produce frequent gaze shifts between visual referents and communicative partners during interaction, and do so in ways that differ from their speech-exposed peers (Lieberman et al., 2014). Clearly, children exposed to sign language adapt their attentional resources to support learning language in the visual modality. But what is the starting point? What visual attentional strategies do very young infants bring to the task of acquisition, and how are these then adapted to accommodate language acquisition in each modality?

In designing our measures, we also took advantage of evidence documenting that hearing infants’ vocalizations serve as an index of their social engagement. Infants start to vocalize within their first few weeks, producing reflexive sounds such as coughing, sneezing, and crying. Infants then progressively extend their vocal repertoires, adding cooing and laughing (1–4 months) followed by babbling (5–10 months; Oller, 1978; Nathani et al., 2006). Hearing infants are sensitive to how their caregivers respond to babbling; when caregivers respond contingently to their babbling, infants adapt their own vocalizations to match the structure of their caregiver’s utterances (Goldstein et al., 2003; Goldstein and Schwade, 2008).

Young hearing infants are also attuned to how their own vocalizations serve as a means of engaging others. For example, infants’ reactions during the still-face paradigm document that they systematically increase their own vocalizations in an attempt to re-engage a communicative partner who stops interacting with them (Delgado et al., 2002; Goldstein et al., 2009). Hence, infant vocalizations can be a powerful indicator of their engagement with a social partner within an interactive turn-taking communicative context. At issue is whether “sign-naïve” infants appreciate the communicative potential of sign language, producing vocalizations to engage a communicative partner who signs, just as they engage a communicative partner who speaks.

In the current study, we ask how 4- to 6-month-old sign-naive infants deploy their visual attention and vocal responses as they view a series of images, along with a woman who indicates each image either in English or in ASL. This design, which builds upon (Novack et al., 2021), permits us to compare how infants divide their visual attention between a communicative partner and an object, across modalities. It also permits us to assess how infants use their own vocalizations to respond to social partners communicating in different modalities. Finally, we examine infants’ vocalizations in two distinct phases: an active phase (when the woman is actively engaged, labeling objects, looking back and forth between the objects and the infant) versus a still phase (when she pauses all activity, casting her glance downward).^1^ Comparing infants’ vocalizations across these phases permits us to ask whether infants are sensitive to the turn-taking episodes of communicative behavior. Based on prior work, we expect that infants in the spoken language condition will vocalize more in the still phase than the active phase (Delgado et al., 2002; Goldstein et al., 2009). It is an open question as to how infants will respond in the sign language condition. If sign-naïve infants appreciate the communicative potential of sign language, they too should vocalize more in the still phase than the active phase. However, it is also possible that sign-naïve infants do not recognize the communicative potential of sign language; if this is the case, they should not vocalize more in the still phase.

## Materials and Methods

### Participants

Participants included 45 infants between the ages of 4 and 6 months (range = 4.05–6.97). There were 23 infants (12 females, *M*_age_ = 5.48, SD_age_ = 0.86) in the sign language condition and 22 infants (13 females, *M*_age_ = 5.37, SD_age_ = 1.00) in the spoken language condition. Infants were recruited from primarily college-educated, white families from the greater Chicago area. All infants were full term, had normal hearing, and were exposed primarily to spoken English at home. The study was approved by the IRB at Northwestern University under the protocol STU00104124.

### Stimuli

**Figure 1:**
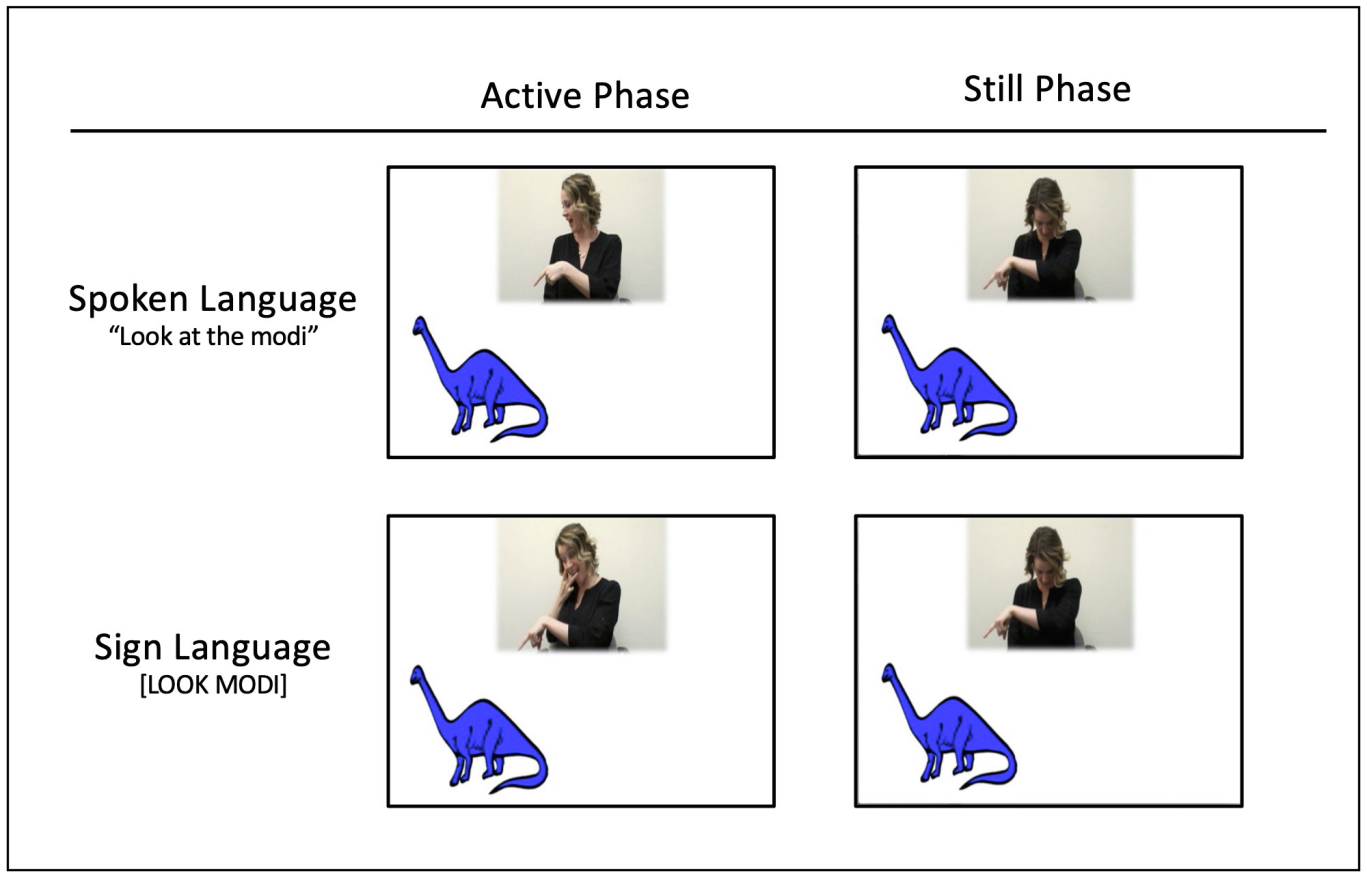
Screenshots depicting one representative trial of the eight trials. In the active phase (left), the woman looks back and forth between the infant and the object while pointing to it and labeling it. This is followed by a still phase (right) in which she ceases all activity, gazing down to break eye-contact. Infants were randomly assigned to condition; in each condition, we counterbalanced (i) whether infants saw a series of images of fish or dinosaurs, and (ii) whether the first image appeared on the right or left side (the image appeared on alternating sides across the eight trials).

Infants viewed a video in which a woman introduced a series of eight exemplars belonging to a single object category. In each trial, a single image (a colored line drawling of either a fish or a dinosaur) appeared on the bottom right or left of the screen; the woman appeared in the top center of the screen. The woman was a hearing, bimodal-bilingual person natively fluent in both ASL and English. To introduce each object, she clapped her hands to attract infants’ visual attention to the screen, then produced an *Active* phase and a *Still* phase (See Figure 1). This sequence was repeated twice for each object.

Active phase (approximately 4,500 ms): The woman looked at, pointed to, and labeled the object. In the spoken language condition, she said: “Look at the Modi. Do you see the Modi?,” using infant-directed speech. In the sign language condition, she signed the phrase, “LOOK MODI, YOU SEE MODI?,” using infant-directed ASL. The pseudo-sign used for MODI was a phototactically well-formed ASL noun (Supalla and Newport, 1978), consisting of two short, straight movements with contact at the cheek, and with a single “8”—handshape. In both conditions, she pointed to and looked at the object while labeling it.

Eye-gaze was identical across the two conditions. The woman looked directly at the infant as she clapped, and then turned to glance at the object as she pointed, saying “look at the Modi/LOOK MODI.” She then turned her gaze back toward the infant, saying “do you see the Modi…/YOU SEE MODI….” As she completed this phrase, she glanced back to the object and pointed to it when she mentioned its name.

Still phase (approximately 3,700 ms). Next, the woman looked down, averting her eye-gaze from the infant and remaining still.

### Procedure

Infants were tested in a quiet room in a university laboratory. Infants sat on their caregiver’s lap approximately 1 meter from a large (115 cm high x 154 cm wide) screen. A hidden video-camera recorded infants’ eye movements and vocalizations. Caregivers wore opaque glasses and were instructed not to interact with their infants during the experiment. Infants saw eight trials in which a woman labeled each object, all from the same category, either in spoken English or ASL. The images (either fish or dinosaur) infants viewed and the side of the first image (right/left) were counterbalanced across participants.

### Behavioral Coding

#### Visual Attention Coding

Trained coders identified infant gaze during each trial, assessing whether the infant was looking on or off screen, and whether the infant was looking toward the woman or the object. Inter-rater reliability, calculated for 1/3 of the participants, was high for both the proportion of on-screen looking (Pearson’s *r* = 0.85, *p* < 0.001) as well as proportion of looking to the woman versus object (*r* = 0.90, *p* < 0.001).

#### Vocalization Coding

Vocalization coding, conducted by an independent set of trained coders, identified any infant vocalizations produced in each trial. Vocalizations that occurred within 1,000 ms of each other were coded as a single unit. For each vocalization, coders recorded whether it was produced in the *active* or *still* phase. Videos from two infants in the spoken language condition could not be coded for vocalizations. Reliability was calculated for 1/3 of the participants. Agreement on whether there was a vocalization in each video phase averaged 97% across all trials.

## Results

### Visual Attention

Infants in both conditions were highly attentive and engaged throughout the task. Those in the sign language condition looked for 80% (SD = 10%) of the total time, whereas infants in the spoken language condition allocated even more attention, looking for 92% (SD = 5%) of the total presentation, *t*(44) = 5.083, *p* < 0.001.

To assess patterns and division of visual attention, we calculated infants’ preference for the woman by dividing their total looking to the woman by their total combined looking to the woman or the object. We then ran a mixed ANOVA on infants’ proportion of attention to the woman with condition (spoken and sign) as a between-subject’s variable, phase (active and still) as a within-subject’s variable, and age as a covariate. The analysis revealed main effects of both condition, (*F*(1,42) = 12.42, *p* = 0.001) and phase (*F*(1,42) = 124.780, *p* < 0.001), qualified by a condition by phase interaction *F*(1,42) = 8.10, *p* = 0.007. There was also a significant effect of age (*F*(1,42) = 8.96, *p* = 0.005), indicating that with age, infants devoted more attention to the woman.

**Figure 2:**
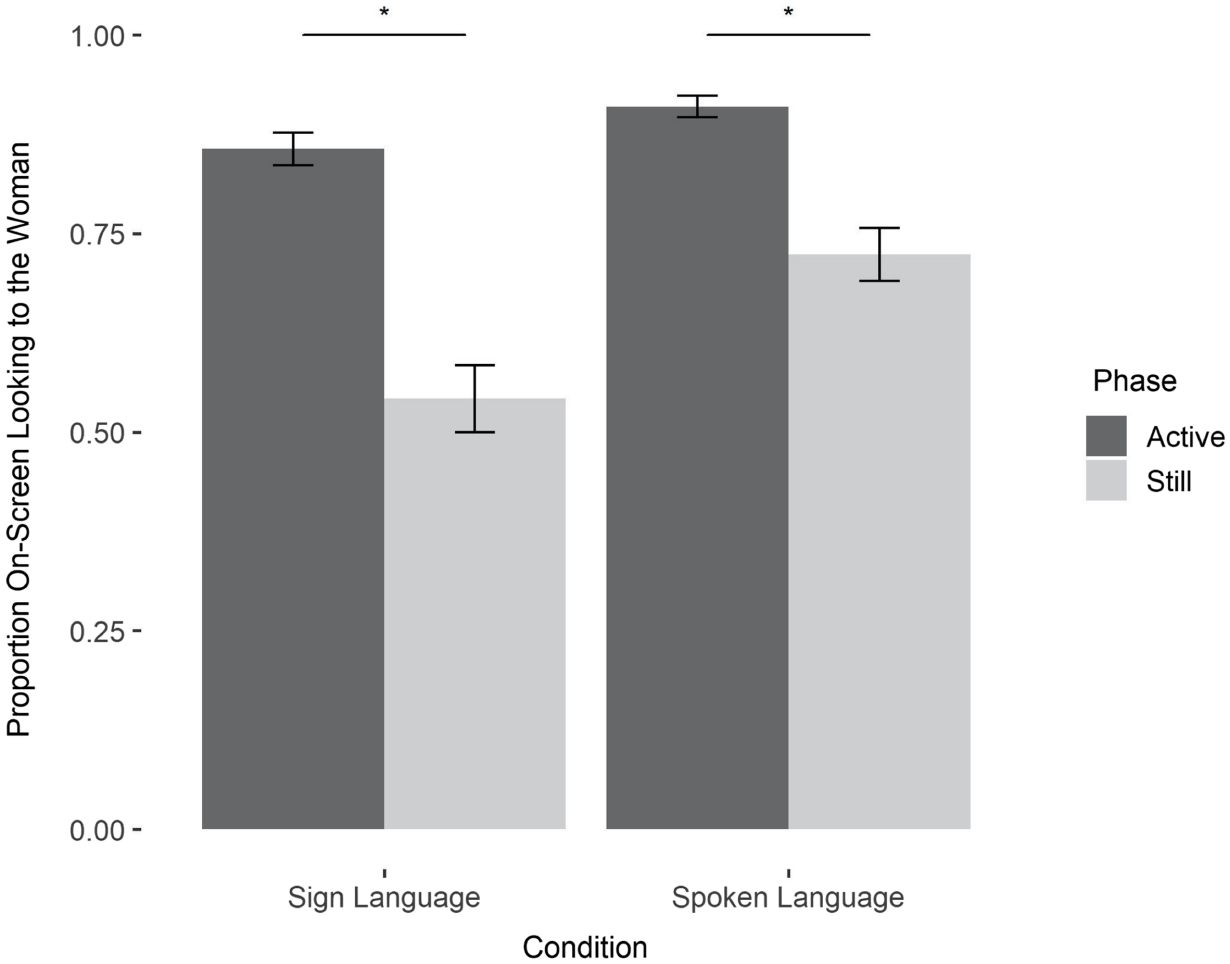
Proportion of on-screen looking to the woman (as compared to the object) by condition and phase. Across both conditions, infants looked at the woman more during the active phase than the still phase (sign: *M*_active_ = 85% SD_active_ = 10%, *M*_still_ = 54%, SD_active_ = 20%, *t*(22) = 9.08, *p* < 0.001; spoken: *M*_active_ = 91% SD_active_ = 6%, *M*_still_ = 72%, SD_active_ = 16%, *t*(21) = 6.53, *p* < 0.001). This difference between active and still was greater for the sign language condition than the spoken language condition (interaction: *p* < 0.001).

The condition by age interaction is depicted in Figure 2. Infants in both conditions devoted more visual attention to the woman than the object; and more to the woman when she was actively communicating than when she was still. Interestingly, the relative difference in attention to the woman varied as a function of condition: infants in the spoken language condition were quite vigilant, focusing predominantly on the woman even in the still phase; infants in the sign language condition were more likely to disengage from the woman when she was still, an outcome that permitted them to devote more attention to the object.

To test the possibility that infants in the spoken language condition were indeed more vigilant to the woman, we tallied the number of times each infant shifted their visual attention between the woman and the object (following analyses in Lieberman et al., 2014). We found that infants’ tendency to switch their visual attention between the woman and the object (during any phase) varied as function of language modality: Infants in sign language condition switched significantly more times than did infants in the spoken language condition (sign: *M* = 5.40 switches, SD = 1.38, spoken: *M* = 4.26 switches, SD = 1.84, *t*(1,43) = 2.359, *p* = 0.02).

### Vocalizations

Most infants vocalized at least once (*N*_sign_ = 19, *N*_spoken_ = 11). On average, infants in the sign language condition produced 4.96 (SD = 4.99) vocalizations, and infants in the spoken language condition produced 2.85 (SD = 4.51), which was not different by condition, *t*(41) = 1.5, *p* = 0.2.

**Figure 3:**
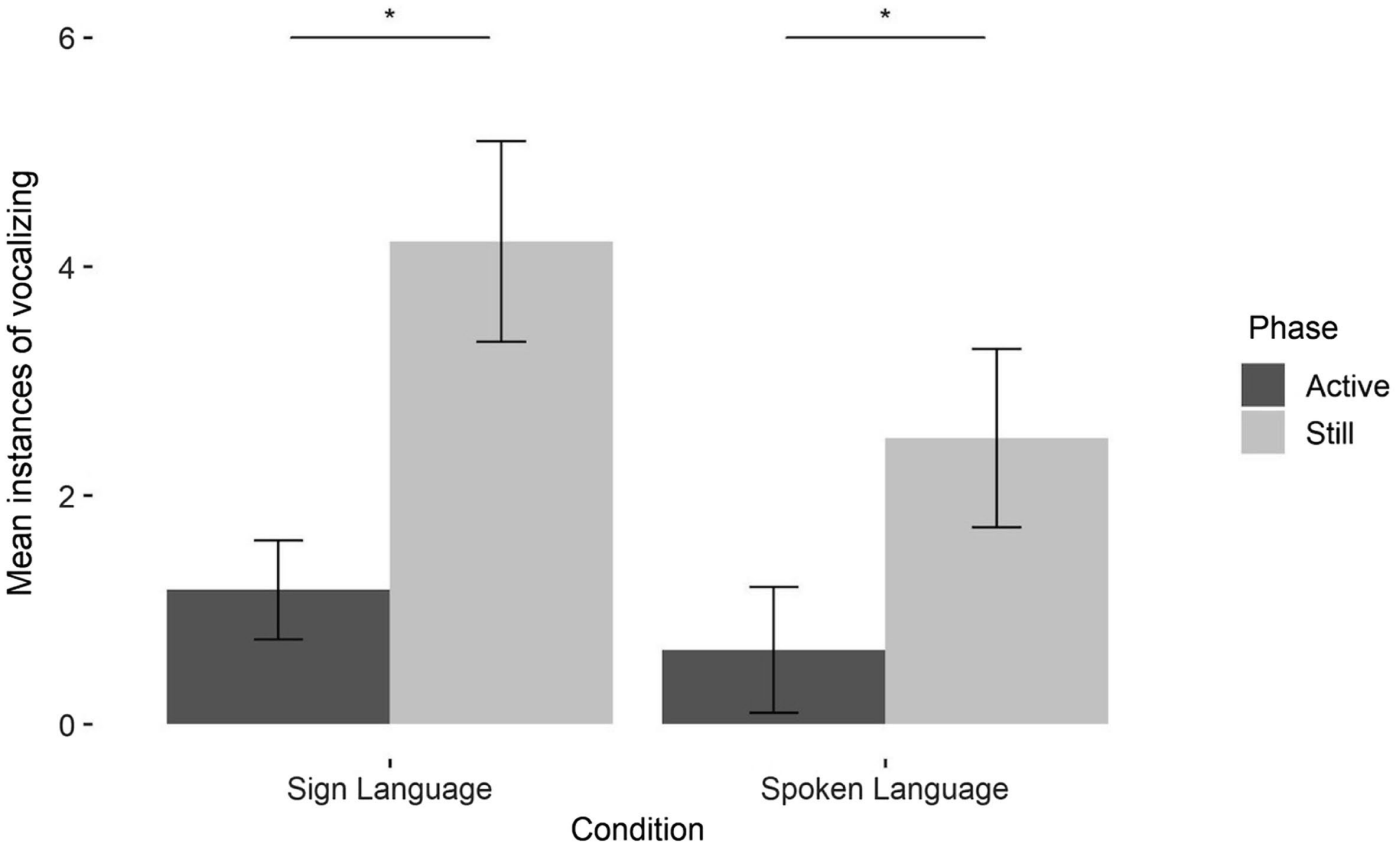
Vocalization production by condition and phase. Within both conditions, more vocalizations occurred during the still phase than the active phase (sign: *M*_active_ = 1.17, SD_active_ = 2.08, *M*_still_ = 4.22, SD_still_ = 4.21, *t*(22) = 3.5, *p* = 0.002; spoken: *M*_active_ = 0.7, SD_active_ = 2.45, *M*_still_ = 2.55, SD_still_ = 3.46, *t*(19) = 3.1, *p* = 0.005).

We tallied, for each infant, all instances of vocalizations that occurred in either the active or still phases. We submitted this to a generalized mixed effect model with phase (active and still) and condition (spoken and sign) as fixed effects, participant as a random effect, and age as a covariate. There was a significant main effect of phase; as expected, infants vocalized more during the still phase (*M* = 3.42, SD = 3.94) than the active phase (*M* = 0.93, SD = 2.25; *β* = 1.28, SE = 0.22, $\chi^{2}$ (2) = 65.1, *p* < 0.0001). Indeed, vocalizations during the active phase were rare in both conditions (Figure 3, dark bars). There were no other significant main effects or interactions (*p*s > 0.1). Thus, 4- to 6-month-old hearing infants appear to be responsive to the communicative value of sign language, restricting their vocal responses to the breaks in communication, just as they do in response to spoken language.

## Discussion

Human language not only engages infants from birth, but also affords powerful conceptual advantages. In the first few months of life, infants’ engagement with language provides the foundation for establishing a link between language, both spoken and sign, and core cognitive capacities such as object categorization (Perszyk and Waxman, 2018; Novack et al., 2021). The goal of the current study was to advance the evidence by assessing how 4- to 6-month-old sign-naïve infants deploy their visual attention and social-communicative strategies in the context of episodes involving either spoken or sign language.

Our findings reveal both commonalities and differences in infants’ responses to spoken and sign language. First, whether they were presented with spoken English or ASL, infants directed their visual attention predominantly to the woman during the active phase. Yet when the woman stopped communicating during the still phase, infants’ performance between the two conditions differed: those in the spoken language condition were more likely to continue to gaze at the woman than were those in sign language condition. This difference during the still phase may reflect infants’ language experience: we suspect that because they have had more exposure to spoken English than to ASL, hearing infants are more strongly motivated to attend vigilantly to a partner who communicates through speech. What remains unknown is whether infants’ vigilance in the spoken condition reflects their greater exposure to English in particular, or to any language presented in the acoustic modality. In future work, it will be important to address this question.

Second, infants in both conditions produced more vocalizations when the woman was still than when she was actively communicating. This increase in vocalizations during the still phase is consistent with the possibility that infants were trying to re-engage the woman or bid her back. Together, these outcomes accord well with the hypothesis that 4- to 6-month-old hearing infants, never before exposed to sign, appreciate the communicative status of *both* spoken and sign language. It also aligns with evidence suggesting that young infants recognize the linguistic potential of language across modalities (Baker et al., 2006; Krentz and Corina, 2008; Palmer et al., 2012; Stone et al., 2018).

These findings also offer a new perspective for investigating infants’ language acquisition across modalities. In particular, the visual presence of the woman producing language is far from trivial. Certainly, her presence on screen was required for the sign language condition. But we found that infants devoted considerable visual attention to the woman both in the sign language condition (when they *had* to look at her to glean language information), as well as in the spoken language condition (when they could have devoted their visual attention to the object). Infants’ responses in the sign language condition offer insight into how they deploy their patterns of visual and social-engagement in a “looking-while-looking” task, in which the objects and linguistic information are both presented to the visual system. This provides an important counterpoint to the more standard ‘looking-while-listening’ tasks, in which objects are presented to the visual system and linguistic information is presented in an auditory stream (e.g., Ferry et al., 2010, 2013; Bergelson and Swingley, 2012; Fernald et al., 2013).

Our findings with sign-naïve infants contribute to recent research testing sign-exposed children in language learning tasks (MacDonald et al., 2020; Lieberman et al., 2021). The distinct attentional responses to language in different modalities, observed here in early infancy, must be independent of language exposure, but may still lay a foundation for the later strategies that emerge specifically for sign-exposed children. In future work it will be important to explore how these patterns emerge and change across development, and in response to different language environments.

It will also be important in future work to address some limitations in the current design. One limitation is that here, we have examined only a single spoken language (English) and a single sign language (ASL). At issue is how broadly these effects hold and how they are mediated by language familiarity and language modality. Another limitation is our reliance on infant vocalizations as an index of social engagement. Certainly this focus on infant vocalizations is well-motivated, but it will also be important to consider infant behavior more broadly, examining for example their motor behaviors as an index of their social engagement. For example, it will be fascinating to assess whether sign-naïve infants attempt to imitate components of the signer’s hand movements. Third, it will be important to delve more deeply into infants’ responses to the woman, comparing their responses documented here to their responses when interacting with a “live” woman. We presented video-recordings because our goal was to present the same woman (a native bi-modal bi-lingual speaker of English and ASL) to all infants. This decision was motivated by strong evidence that 4- to 6-month-old Western-raised infants respond to and understand social communicative interactions from video recordings (e.g., Senju and Csibra, 2008; Lewkowicz and Hansen-Tift, 2012; Liberman et al., 2021), as they did here. But in future work, it will be important to assess infants’ behavior with communicators that are physically present.

Finally, to capture the early attentional and social capacities that infants bring to the language acquisition process, we focused on hearing infants with no prior exposure to sign language. However, it is also important to ask these questions with sign-exposed infants, as well as infants exposed to both sign and spoken language (bi-modal bilinguals). ASL-exposed infants have been shown to demonstrate enhanced gaze control and gaze following as a result of their early visual language experience (Brooks et al., 2020; Bosworth and Stone, 2021). Comparing their attentional patterns to those of sign-native infants will further elucidate the ways in which infants adjust their attentional processes on the basis of their exposure.

The current evidence, which sheds new light on how very young infants allocate their visual attention and engage with communicative partners across different modalities, advances our understanding of the tools infants bring with them to the language learning process and the flexibility with which they deploy them in responding to diverse language experiences.

## Data Availability Statement

The original contributions presented in the study are included in the article/Supplementary Material, further inquiries can be directed to the corresponding author.

## Ethics Statement

The studies involving human participants were reviewed and approved by Northwestern University IRB. Written informed consent to participate in this study was provided by the participants’ legal guardian/next of kin. Written informed consent was obtained from the individual(s) for the publication of any identifiable images or data included in this article.

## Author Contributions

MN, DC, and SW contributed to the design and implementation of the research methods, coding, data analysis, and writing of the manuscript. All authors contributed to the article and approved the submitted version.

## Funding

This research was made possible by the following grants from NICHD: R01HD083310 to SW and F32HD095580 to MN.

## Conflict of Interest

The authors declare that the research was conducted in the absence of any commercial or financial relationships that could be construed as a potential conflict of interest.

## Publisher’s Note

All claims expressed in this article are solely those of the authors and do not necessarily represent those of their affiliated organizations, or those of the publisher, the editors and the reviewers. Any product that may be evaluated in this article, or claim that may be made by its manufacturer, is not guaranteed or endorsed by the publisher.

## Supplementary Material

The Supplementary Material for this article can be found online at: https://www.frontiersin.org/articles/10.3389/fpsyg.2022.896049/full#supplementary-material

## Footnotes

^1^Note, the current study methods differ somewhat from the prior published work (Novack et al., 2021), which used slightly different stimuli. Novack et al. (2021) compared sign language to non-linguistic pointing, here we compare sign language to spoken language. Additionally, stimuli in the version used by Novack et al. (2021) had the woman fade out of view between communicative episodes. Here, the woman remains on screen the entire time.
